# Darwin’s perception of nature and the question of disenchantment: a semantic analysis across the six editions of *On the Origin of Species*

**DOI:** 10.1007/s40656-021-00373-y

**Published:** 2021-04-14

**Authors:** Bárbara Jiménez-Pazos

**Affiliations:** 1 IAS-Research - Centre for Life, Mind and Society. Philosophy Department, University of the Basque Country, San Sebastián, Spain; 2 Facultad de Educación, Filosofía y Antropología, Universidad del País Vasco, Avenida de Tolosa 70, 20018 San Sebastián, Guipúzcoa, Spain

**Keywords:** Darwin, The Origin of Species, Disenchantment of the world, Perception and description of natural beauty, Computer-assisted lexical analysis, Semantic analysis

## Abstract

This body of work is motivated by an apparent contradiction between, on the one hand, Darwin’s testimony in his autobiographical text about a supposed perceptual colour blindness before the aesthetic magnificence of natural landscapes, and, on the other hand, the last paragraph of *On the Origin of Species*, where he claims to perceive the forms of nature as beautiful and wonderful. My aim is to delve into the essence of the Darwinian perception of beauty in the context of the Weberian concept of “disenchantment of the world”, assumed as a possible conceptual axis that enables the unravelling of the core of this contrast of perceptions. In acknowledging the theory of evolution as one of the most prominent scientific theories likely to have contributed to disenchantment, a number of questions arise: Is disenchantment compatible with aesthetic experience and sensibility before natural beauty? Was it Darwin’s disenchanted conception of the world that led him to believe he was colour blind? To answer these questions, a computer-assisted semantic analysis of lexical frequency and variability, most especially focused on aesthetic-emotional and religious or spiritual adverbs and adjectives, has been undertaken across the six editions of *The Origin*. The semantic analysis demonstrates that, although disenchanted, Darwin’s descriptions of, mainly, the adaptational excellence of living beings, reflect an aesthetically enriched perception of nature. It is concluded that Darwin’s perceptual colour blindness, then, might be based on a confusion rooted in the equation of equality between aesthetic sensibility in nature and the perception of its beauty as part of the *vestigia Dei*.

**Supplementary Information:**

The online version of this article (10.1007/s40656-021-00373-y) contains supplementary material, which is available to authorized users.

## Introduction

The last paragraph of Charles Darwin’s *On the Origin of Species* (1859) (hereinafter OS) is memorable for it masterfully synthesises the work’s content through an aesthetically and ontologically optimistic language of Humboldtian demeanour:^1^

It is interesting to contemplate an entangled bank, clothed with many plants of many kinds, with birds singing on the bushes, with various insects flitting about, and with worms crawling through the damp earth, and to reflect that these elaborately constructed forms, so different from each other, and dependent on each other in so complex a manner, have all been produced by laws acting around us. […] There is grandeur in this view of life, with its several powers, having been originally breathed^2^ into a few forms or into one; and that, whilst this planet has gone cycling on according to the fixed law of gravity, from so simple a beginning endless forms most beautiful and most wonderful have been, and are being, evolved (Darwin 1859, pp. 489**–**490).

Darwin shares his view of life, described as grandiose (“There is grandeur in this view of life”), made up of an admirable interconnection between laws and beautiful and wonderful organic beings (“endless forms most beautiful and most wonderful”); this paragraph transmits, without a doubt, a message about the intellectual, sentimental and aesthetic appeal that Darwin takes on from the study of nature. Surprisingly, these emotionally inspiring words have remained almost unchanged from the first drafts of OS,^3^ which contain the ancestor versions of Darwin’s concluding lines (Darwin (ed.) 1909a, 1909b).

The aesthetic and ontological optimism that is inferred from these final lines lies in Darwin’s ability to observe nature through the optics of his theory of evolution and, consequently, perceive its beautiful and wonderful forms in slow but constant development. Darwin’s aesthetic-ontological optimism is also, therefore, disenchanted, in the strict sense of the term, that is, desacralized, or of an evidently naturalistic inclination, since there are no indications to understand Darwin’s explanation of the existence and evolution of species as cases of *vestigia Dei*.

Whatever this view of disenchanted and optimistic life, it is contrasted with the regretful confession Darwin makes in his autobiographical text^4^ (hereinafter AB) about a supposed loss of the ability to perceive intense aesthetic-religious feelings in nature, which had remained active in his youth. In this regard, Darwin says he feels colour blind in the face of great scenes of nature:

I well remember my conviction that there is more in man than the mere breath of his body. But now the grandest scenes would not cause any such convictions and feelings to rise in my mind. It may be truly said that I am like a man who has become colour-blind, and the universal belief by men of the existence of redness makes my present loss of perception of not the least value as evidence (Barlow 2005, p. 76).

This confession, belonging to a section on Darwin’s “Religious Belief” (Barlow 2005, pp. 71–80), reveals the interruption of the interrelation between the experience of religious feelings, in the face of the magnificence of nature, and Darwin’s capacity for aesthetic perception in such a way that the former cannot occur without the later. It could be said that, in contrast with what can be inferred from the last lines of OS, Darwin’s confession reveals the pessimistic consequences of assuming a disenchanted view of life.

Could what Darwin diagnoses as “loss of perception” be related with, as he adds later in the text, a loss of his capacity for artistic enjoyment? While Darwin’s fascination for scientific content in any text had remained alive, interest in music, poetry and landscape observation had disappeared (Barlow 2005, pp. 112–113). The unconfirmed diagnosis Darwin himself makes about his artistic discomfort is certainly suggestive; he contemplates the possibility that, over time, an over-dedication to the scientific study of nature may have atrophied, due to disuse, the part of his brain dedicated to the appreciation of the finest arts (Barlow 2005, p. 113).

Could both losses, —the aesthetic and religious perceptual inability for natural magnificence and the disappearance of artistic enjoyment—, be attributed to the same cause? Both the symptoms and the diagnosis could point to a possible Weberian disenchantment—explained in more detail below in Chapter 2—likely caused, to some extent, by Darwin’s assimilation of his own scientific ideas and a consequent loss of the meaning of life. The side effect of scientific progress and the world’s demystification, for Weber, is that the world becomes intellectualized, rationalized, predictable and thus, disenchanted, but also disenchanting, as a consequence of the scientific incapacity to fill the vacuum left by the decline of religion. The world, in sum, becomes undesirably unable to fulfil emotional needs.

Weber’s diagnosis of the disenchantment of the world could be a useful way to rephrase what Darwin experienced, since it could explain the artistic and aesthetic-perceptual blindness he described in AB. But it is paradoxical that Darwin ends OS, a disenchanting milestone in the history of science, with words that appeal to the grandeur of nature and convey positivity and intellectual, aesthetic and sentimental satisfaction, while it is not until late publication of AB that he reveals the negative effects of an alleged disenchantment, in the Weberian sense of the term.

In short, on the one hand, the last lines in OS show a clear optimistically disenchanted aesthetic sensibility arising from various sources of inspiration such as the awareness of a vital activity that is infinite in appearance and perfect in essence. On the other hand, the testimony in AB about Darwin’s “atrophy” in one part of the brain and an alleged “colour blindness” would indicate, consequently, a feeling that does not seem to correspond to what was expressed in the last paragraph of OS.

The ideas just described then point to a contradiction that allows the formulation of questions that will mark the argumentative development of this article. If Darwinian science,—whether it is an over-dedication to scientific study, or perhaps the internalization of a supposedly pessimistically disenchanting message found in OS—, is the cause, according to Darwin, of the atrophy of the part of his brain dedicated to artistic appreciation, and also of the inability to experience feelings of aesthetic exaltation in nature, why does Darwin end OS, one of the most outstanding works in the history of science that would have contributed to disenchantment, with a message that indicates intellectual and aesthetic fascination? Is disenchantment compatible with aesthetic experience and sensibility for natural beauty? Considering that the OS text varied considerably over the years, leading to different editions with multiple additions and deletions, would it be possible to detect in the lexical-argumentative evolution of the different editions of OS signals that anticipate a late disenchantment in Darwin? Is it his disenchanted conception of the world that led Darwin to believe himself colour blind in the face of the beauty of nature? Was Darwin disenchanted at the end of his life, or just confused by his inexplicable lack of aesthetic and artistic interest? I will answer these questions in four chapters dedicated to unravelling the characteristics of Darwin’s perception of natural beauty.

To start with, the following chapter will define the conceptual pillars of this article around the notion of Weberian disenchantment, and its relationship with Darwin’s work, through a critical review of the state of the art. The review of the literature will, consequently, allow us to propose the working hypothesis and justify the methodology that I have used to solve the dilemma about Darwinian disenchantment.

## Disenchantment: hypothetical and methodological remarks

One of the cultural manifestations that came out of the historical process of the “desacralization” or the “secularization”^5^ of the world, is the “disenchantment of the world” (*Entzauberung der Welt*),^6^ proposed by Max Weber in his 1917 lecture “Science as a Vocation” (*Wissenschaft als Beruf*), a phenomenon of modern society that arises from cultural intellectualization and rationalization. Weber attributes the emergence of the disenchantment of the world to the possibility of access to a rationalized knowledge of our living conditions:

The growing process of intellectualization and rationalization does *not* imply a growing understanding of the conditions under which we live. It means something quite different. It is the knowledge or the conviction that if *only we wished* to understand them we *could* do so at any time. It means that in principle, then, we are not ruled by mysterious, unpredictable forces, but that, on the contrary, we can, in principle, *control everything by means of calculation*. This in turn means the disenchantment of the world. Unlike the savage for whom such forces existed, we need no longer have recourse to magic in order to control the spirits or pray to them. Instead, technology and calculation achieve our ends. This is the primary meaning of the process of intellectualization (Weber 2004, pp. 12–13).

From Weber’s words it can be inferred that the concept of the “disenchantment of the world” is a clear expression of how the characteristic type of knowledge of modern science affects the view and emotional perception of the world in specific cultural contexts. In the case of the modern cultural context to which Weber refers to, the detachment of assumptions about the functioning of the world based on mysterious forces caused by trust in technology and calculation would have been the essence of disenchantment.

In this regard, it should be borne in mind that the Weberian diagnosis of the disenchantment of the world caused by modern science is only a generic description of a type of modifications that the history of science has introduced in the worldview and in cultural consciousness; these modifications have crystallized in processes categorised as “secularization” or “desacralization” of the world and culture. Lately, these processes are being taken up in contemporary philosophical historiography under partially equivalent descriptors such as “metaphysical naturalism” and “scientific naturalism”.

A specific case of the relationship between scientific knowledge and culture that would confirm the Weberian thesis on the disenchantment of the world is, precisely, the publication of Darwin’s OS. As we will discuss in more detail in Sub-chapter 2.1., the bitter cultural reception of the secularizing implications of the analysis of nature that Darwin proposed in OS has been, in addition, one of the preambles of a wave of social analysis about why science would not be oriented to, nor would it be able to, make sense of the world. As an example of this, a number of studies have, in fact, managed to perpetuate the idea that modern science would consequently result in the irreversible dehumanization of culture (see, for instance, Adorno and Horkheimer 2016).^7^

### Disenchantment and Darwinian disenchantment

Darwin’s OS is certainly considered a paradigmatic work in the history of science because of the disenchanting effect caused by the cultural reception of its ideas, both in the strict lexical sense of the term “disenchantment”, and in the pessimistic sense most aligned with the emotional state of all those who internalize the ideas developed in the work.

Regarding the strict sense of the term “disenchantment”, it should be noted that the argumentative axis of OS, the theory of evolution, explains the functioning of the mechanism that leads to the evolution of species, natural selection, casting aside the need to appeal to supra-natural—enchanted—entities. That is, Darwin undid the need to believe in a Creator of—independently created ad hoc—animal species and laid the groundwork for the belief in a progressive evolution of species, which included human beings, from a common ancestor.

As for the emotionally pessimistic, or pejorative, sense of the term “disenchantment”, it is the perspective that assumes that the enchantment of nature, that is, its aesthetic and emotional appeal, deteriorates as science explains its secrets. To this respect, the biological—the discovery of an ancestor of human beings, which were to be inserted as another link in the evolutionary chain—and, above all, the cultural implications resulting from the assimilation of the disenchanting conclusions that Darwin raised in OS should be remembered. These ideas acted as the disconnectors of enchantment, generating a feeling of social unrest moved by the loss of the meaning of life, in the cultural context of that time. Accepting Darwinian science required eliminating fundamental cultural presuppositions related to religious morals.

One of the most manifest analyses of both the strict and pessimistic senses of Darwinian disenchantment is that of D. C. Dennett (1995). This author presents “Darwin’s dangerous idea” as a “universal acid” that corrodes the network of traditional ideas and offers a new revolutionary view of the world based on natural selection being considered to be a mechanical “algorithm” that dispenses with an intrinsic and controlling rationality of its own mechanism.

The recent historiographical studies, a majority of which are reactions to scientific disenchantment in general, and a minority to Darwinian disenchantment in particular, still confirm the feeling of social discomfort. These studies, some more profusely than others, analyse—and many of them also boost—the concepts of “enchantment” and, above all, “re-enchantment” and its multiple manifestations, namely, artistic and literary, ecological, mystical or spiritual, religious and secular or scientific as strategies for re-enchanting the world (Swatos 1983; Berman 1981; Lassman and Velody 1989; Scribner 1993; Kontos 1994; Schroeder 1995; McDowell 1996; Curry 1999; Berger 1999; Ruickbie 1999; Jenkins 2000; Griffin 2001; Gane 2002; McGrath 2002; Owen 2004; Partridge 2004; Koshul 2005; Graham 2007; Levine 2008, 2011; Walsham 2008; Gibson 2009; Landy 2009; Landy and Saler 2009; Paige 2009; Tanaka 2009; Richards 2011; Taylor 2011; Asprem 2014; Josephson-Storm 2017).

As far as Darwinian disenchantment is concerned, R. J. Richards (2011), a defender of Darwinian enchantment whose tendency has been to consider Darwin as an author decisively influenced by romantic naturalism,^8^ and especially G. Levine (2008; 2011), a defender of a Darwinian re-enchantment, are the authors who most profusely have studied the relationship between the concept of disenchantment and Darwin’s work. Levine (2008) suggests that one should not assume the view of nature that Darwin proposes in OS as disenchanting, but as secularly re-enchanting. In this regard, Levine argues that Darwin’s ability to be amazed by the wonders of nature, which is visible, for example, in the last lines of OS, is an obvious sign that there is no reason why the assimilation of the ideas set forth in this work has to produce disenchantment, in the pessimistic sense of the term, and that it could, on the contrary, encourage a secularly re-enchanting view of nature. To prove this, he highlights the supposedly frequent use of the word *wonder*, and its derivative terms, in OS.

Although I do not share Levine’s proposal of considering Darwinian science as re-enchanting, I will adopt his rejection of the position that assumes it as disenchanting, in an emotionally pessimistic sense. I will also adopt the methodology of analysis of lexical frequencies. Nevertheless, unlike Levine’s, my analysis will be computer-assisted and, therefore, quantitatively more exhaustive.

The best and clearest defence of the active knowledge of nature^9^ is the manifest opposition of Alexander von Humboldt in his work *Kosmos*, published in five volumes from 1845 to 1862, to the relationship between the study of nature and the loss of its enchantment. Only by studying the exact sciences, says Humboldt, can the pleasure produced by the contemplation of nature be found (Humboldt 1993, p. 28). This is precisely the point of view on which I have constructed my hypothesis about Darwinian disenchantment in the following sub-chapter.

### Hypothesis

The hypothesis I intend to explore—I will formulate a complementary hypothesis in Chapter 4—is that, contrary to what can be inferred from Darwin’s diagnosis about a perceptual colour blindness, the progressive acquisition of explanatory knowledge about nature should not have weakened Darwin’s aesthetic sensibility, but, on the contrary, it should have refined and matured it, as can be seen from the disenchanted, although aesthetically and emotionally satisfied view of life that Darwin describes at the end of OS. Whereas the theory of evolution certainly divests nature of its magical character in explaining the mechanics of natural selection, it also should have accorded a more profound and completely new understanding of it to Darwin.

A specifically human way, although not the only one, to relate to nature, is in its perception and description, as both are inseparable from the idea of nature in force in each cultural environment. So, regardless of Darwin’s assessment of his own aesthetic sensibility against natural beauty in AB, whether or not he conveys a non-pejorative disenchanted conception of nature should be made visible by examining relevant lexical frequencies and variations in the vocabulary used to describe nature across the six editions of OS (1859, 1860b, 1861, 1866, 1869, 1872). The semantic testimony of the lexicon in the six editions of OS should have more authority than the opinion that Darwin himself could hold at any given time about his own aesthetic sensibility. And, of course, the texts should have more authority than Darwin’s will of the meaning of his own texts, for these reveal, in the end, what their semantic mass contains, including its logical implications, and this need not necessarily coincide with the will of its author.

As the publication of new editions of OS progressed, Darwin eliminated and added a multiplicity of paragraphs, updated hypotheses, refined ideas, corrected explanations and improved descriptions. Therefore, the lexical variability, from the first edition to the sixth, should be one of the most notable indicators of the underlying conceptual structure of OS, which reflects, in turn, the ontological and epistemological presuppositions on which Darwin’s conception of nature is built. The frequency, the modifications and the nature of the terminology, should be illustrative indications of the consequences that the development of the evolutionary theory has had on Darwin’s perception and description of nature and, consequently, on the issue of disenchantment.

Unfortunately, the critical literature around the Darwinian lexicon is not abundant. There are concordances of OS (Barrett et al. 1981) and other works by Darwin (Barrett et al. 1986, 1987; Weinshank et al. 1990), as well as the digital^10^ and printed (Peckham 1959) versions of the *variorum* of the six editions of OS. Other studies, minimal in some cases, have focused on specific aspects of Darwinian lexicology and the evolution of Darwin’s thought across editions (Vorzimmer 1972; Liepman 1981; Sulloway 1985; Loye 2000; Richards 2002, 2011; Sloan 2005; Shillingsburg 2006; Levine 2011; Sainte-Marie et al. 2011; Hidalgo-Downing 2014; Menninghaus 2016; Hoquet 2013, 2018). However, there has been no semantic study of the lexicon that confirms or refutes the thesis of disenchantment in Darwin’s work.

In short, if Darwin’s work had contributed to the disenchantment process, then it should be possible to detect its semantic traces in Darwin’s description and perception of nature. A semantic analysis on lexical frequency and variability should, in turn, clarify, with a greater precision than is available in historiography, the issue of whether the assimilation of Darwin’s own theory weakened or, on the contrary, strengthened his aesthetic sensibility.

### Methodology

To test the just-described hypothesis, I have developed a computer-assisted^11^ study on the Darwinian lexicon, especially focused on the lexical frequency and variability across the six editions of OS. This lexical data analysis should help trace the onto-epistemological presuppositions underlying Darwin’s descriptions of nature.

To answer the question of whether the disenchanted conception of the world weakened Darwin’s aesthetic sensibility, the key terminology that to this respect I have chosen to analyse is mainly the types of adjectives and adverbs related to: 1) descriptions of nature which have an aesthetic bias, as well as; 2) Darwin’s moods with respect to nature, and; 3) general impressions that denote emphatic interest in the knowledge of the natural landscape. The results obtained from the analysis focused on the number of occurrences, as well as on the type and variability of the expressions affected by this category of adjectives and adverbs, should be reliable indicators of the Darwinian capacity for the aesthetic-emotional perception of nature.

It seems reasonable to assume that the use of a lexicon of supra-natural semantics, that is, with an implicit ontology prone to regard nature as a numinous reality not explainable by itself and populated by entities, virtualities or traits of trans-natural descent, is a good indicator of a non-disenchanted worldview in the sense described above.^12^ Therefore, I have studied the frequency and variability in OS of: 4) adjectives—no adverbs have been found—*potentially* categorizable as religious or spiritual, as lexical indicators of ontological presuppositions, in Darwin’s description and perception of nature, incompatible with a hypothetical disenchanted conception of nature. A semantic study focused not only on the presence, absence or variability of religious or spiritual adjectives in OS, but also on the types of nouns affected by these adjectives, and the lexical context in which they have been inserted, should determine the degree of religiousness of these terms and, above all, their relevance for Darwin’s theory.

Obviously, some of the adjectives and adverbs I have considered to be aesthetic-emotional, such as *wonderful*/*fully* o *beautiful*/*fully*, could be considered to be religious if the lexical context in which they were inserted was semantically interpretable as religious.^13^ This is why I insist on the importance of also semantically analysing all the nouns affected by the chosen adjectives and adverbs, as well as the lexical context, since only the semantic analysis of the Darwinian lexicon will determine the nature of the terms.

To carry out an analysis of the Darwinian lexicon through the six editions of OS I have had to computationally process the vocabulary contained in these editions using both manual text-editing and text-mining strategies, and *WordSmith Tools* (Scott 2020), a software package for linguistic corpus analysis. To do this, firstly, since *WordSmith Tools* only processes files in txt format, I have taken as reference the textual versions of the six editions of OS available at http://darwin-online.org.uk (van Wyhe 2002) and, following manual text-editing strategies—these include, for instance, eliminating special characters the software package does not properly process, or reducing the length of the texts by eliminating the space between paragraphs—, I have thoroughly prepared the texts to create six documents, each corresponding to the six editions of OS, in txt format. Secondly, I have processed these six documents with the *WordList* tool in *WordSmith Tools* to extract word frequency lists, which contain all the lexical material of the six editions of OS; in this type of lists, words occurring in the texts are ordered by their frequency of occurrence, from the most commonly occurring words down to those words that appear less frequently. Thirdly, after an in-depth scrutiny of all the words included in the six word frequency lists, I have selected the key adjectives and adverbs that are relevant to this study—such as the ones which meet the conditions just described in points 1, 2, 3 and 4—. Finally, I have manually analysed these words, one by one, in each of the six txt files, in order to, first, know which nouns and verbs they affect and, second, detect word additions, deletions or lexical variations that Darwin might have applied to the different OS editions.

I am aware of the miscalculations that can be made in a manual, not automated lexicon scrutiny. Therefore, to guarantee the exhaustiveness of my analysis on occurrences and lexical variability, I have complemented my manual text-mining strategies with the use of the *Concord* tool in *WordSmith Tools*, useful to locate all the occurrences of a specific word in their textual context.

The comprehensive development of this computational methodology should contribute to determining whether or not the knowledge of the evolutionary principles prompted Darwin to create a de-spiritualized, disenchanted worldview, yet, intellectually and aesthetically more valuable and intriguing.

## Semantic analysis of the lexicon in *The Origin of Species*

As a result of the study of the Darwinian lexicon in OS, I have created two tables and a graph that house the results obtained with the assistance of *WordSmith Tools*. On the one hand, Table 1 (Online Resource, p. 2)^14^ shows the total values of occurrences, in the six editions of OS, of the aesthetic-emotional and religious adjectives and adverbs extracted from the word frequency lists. Those adjectives of a religious or spiritual nature have been highlighted in bold. This way of presenting the results allows, firstly, to easily visualize the number of occurrences of each adjective and adverb, as well as its increasing, decreasing or null evolution throughout the six editions of OS; secondly, it also allows a comparison of the frequency of a specific adjective or adverb with those other words in the list. Additionally, I have created Graph 1 (Online Resource, p. 3),^15^ which illustrates the frequency results included in Table 1 and, therefore, facilitates the visualization of the lexical conduct of the terms across the different editions of Darwin’s work.

On the other hand, Table 2 (Online Resource, pp. 4–11)^16^ collects the expressions, composed mainly of nouns and verbs, affected by the aesthetic-emotional and religious—also highlighted in bold—adjectives and adverbs. This type of table facilitates a semantic analysis of the results, as it shows the nouns or verbs affected by the selected adjectives and adverbs, as well as the possible addition of new nouns or verbs, their subtraction or permanence throughout the several editions of OS. The terms Darwin removes are preceded by the subtraction symbol “−” and crossed out. The terms he adds appear preceded by the addition symbol “+”. The terms subject to some kind of lexical modification are underlined and they appear separated from the main lexical results, since they are not part of the calculation of occurrences, and these are only highlighted to indicate that they have been modified. Cases in which no expressions affected by a specific adjective or adverb have been found have been indicated with a simple hyphen (-).

This leads to my semantic analysis, in the following three sub-chapters, of the results presented in Table 1 and Table 2. It should be noted that the semantic value of both tables is equivalent. The difference between the two is the visual display of the lexical results. While Table 1 only shows the numerical values corresponding to the occurrences of each aesthetic-emotional and religious adjective and adverb in the six editions of OS, Table 2 also displays all the expressions affected by the adjectives and adverbs.

Thus, Sub-chapter 3.1. will outline the results obtained from a semantic analysis of the terms affected by the aesthetic-emotional adjectives and adverbs. Sub-chapter 3.2. will address the semantics of a religious-type adjectives.

### Semantic analysis of aesthetic-emotional lexicon in OS

The linear display of results in Table 1 (Online Resource, p. 2) allows the easy detection of the aesthetic-emotional adjectives and adverbs with the highest frequency of occurrence in OS. The following terms are mentionable in this respect, as they appear at least five times in most editions of OS: *Admirable*, *astonishing*, *attractive*, *beautiful*, *beautifully*, *extraordinary*, *extraordinarily*, *marvellous*, *prodigious*, *sweet*, *wonderful* and *wonderfully*.

Based on the results shown in Table 2 (Online Resource, pp. 4–11), in Sub-chapter 3.1.1. I will semantically analyse the expressions affected by the most frequently used aesthetic-emotional adjectives and adverbs. Sub-chapter 3.1.2. will focus on the semantics of the terms affected by adjectives and adverbs with less occurrences.

#### Frequent aesthetic-emotional adjectives and adverbs in OS

A semantic analysis of the most frequent aesthetic-emotional adjectives and adverbs shown in Table 2 (Online Resource, pp. 4–11) reveals that the scientific research of nature is the way through which Darwin experiences intense aesthetic feelings and intellectual pleasure. These emotions, acquiring an increasing degree of aesthetic maturity and scientific sophistication across the editions of OS, especially derive from the specialized study of the natural landscape, a study that refines Darwin’s ability for aesthetic and intellectual appreciation of the biological complexity and adaptational perfection and excellence of nature. This is manifested, without prejudice to occasional allusions to the beauty of the visually perceptible characteristics of natural forms and beings, in descriptions of nature that go beyond the aesthetic-emotional description of the merely visual. Instead, Darwin focuses on facts, changes, the functional, structural and instinctive excellence of living beings, powers and invisible or unknown natural mechanisms. The prevalent use of aesthetic-emotional adjectives and adverbs to describe physical or biological aspects of nature, could be considered an indication of an optimistically disenchanted type of lexicon, that is, emotionally suggestive despite its markedly naturalistic inclination.

Among the lexical results that best manifest these conclusions,^17^ the spectrum of expressions affected by the adjective *beautiful*, as well as their evolution through OS editions, have a high semantic value. The lexical results show that Darwin, for the most part, describes as beautiful, from the first to the third edition of OS, aspects of nature such as *blue colour* (*bird*), *races of plants*, *co-adaptations* (2 times), *adaptation/s* (6 times), *diversity and proportion of kinds*, *males*, *contrivance* (2 times), *ramifications*, (*and harmonious*) *diversity of nature*, *work* (bees’), *endless forms*, (*really wondrous and beautiful*) *organisation* or (*and complex*) *structure*. Darwin’s lexicon shows that his descriptions of natural beauty mostly focus on structural, organisational, or distributional aspects of nature such as a biological being’s ability to adapt, the diversity of nature or the functional organisation and structure of some living beings. That is, Darwin not only appreciates the visual beauty of natural objects, but, above all, he appreciates the beauty of technical complexity, functional excellence and the diversity of natural mechanisms.

The functional excellence of nature is equivalently accentuated with the use of the adverb *beautifully*. The expressions that most stand out are those referring to the beauty —which could also be considered “elegance”— found in the perfection of physical structures and, above all, to the ability of living beings to adapt to certain living conditions: *beautifully adapted to its end* (structure of a comb), *beautifully constructed natatory legs*, *beautifully adapting* (power), *beautifully related to complex conditions of life* (parts of organic beings), *beautifully adapted* (giraffe’s frame) and *beautifully adapted* (structures).

It must not go unnoticed that, in the fourth edition of OS, Darwin adds a multiplicity of nouns described as *beautiful*, mostly referring to objects of nature, such as *crystalline lens*, *organic beings*, *objects* (3 times), *volute and cone shells*, *productions of nature* (flowers), *male animals*, *birds*, *fishes*, *mammals*, *butterflies*, *insects*, *reptiles*, *males*, *colours* (2 times, once eliminated in the 5th ed.), *flowers* (2 times), *fruits* (4th–5th eds.), or *living objects* (4th–5th eds.). Nevertheless, the inclusion of this extensive set of terms in the fourth edition shows the influence of a background of purely naturalistic interest. For instance, we can highlight examples that, on the one hand, indicate a strong influence of a base of physical knowledge about nature (*beautiful crystalline lens*, *beautiful volute and cone shells*) and, on the other hand, they occasionally refer, in the framework of explanations about sexual selection or the pollination of plants, to aspects that other organic beings, not Darwin—as it also happens with the adjectives *attractive* and *sweet*—, find beautiful, such as *colours*, *fruits* or *flowers*. This lexical fact leads us to think that a greater acquisition of specialized knowledge about nature makes it possible for Darwin to provide a more complete, specific, refined and detailed set of organic aspects or objects that make up the natural landscape, as well as the corresponding occasional aesthetic qualification.

The fact that in a work like OS the adjective *beautiful* has a considerable lexical presence that increases throughout the editions—this is confirmed by the fact that there is a terminological increase of more than twice as many occurrences from the first edition of OS to the sixth—, not only allows us to confirm that Darwin’s aesthetic interest increases in line with his growing scientific knowledge of nature, but points out that Darwin does not want to dispense with descriptions that show his aesthetic and emotional appreciation of the objects of study. Why would Darwin need to include his aesthetic-emotional assessment of the mechanisms and processes that he explains, if not to assert his aesthetic and intellectual fascination?

Likewise, the adjective *wonderful*, which is the most frequent aesthetic-emotional adjective in OS, positively reinforces the conclusions reached so far, as it affects, from the first to the third edition of OS, a vast number of nouns such as *difference in beaks*, *development*, *fact/s* (6 times), *structure* (the eye) (1st–5th eds.), *power of scent*, *metamorphoses in function* (1st–5th eds.), *instinct/s* (8 times), (*not very wonderful*) *instincts*, (*not very wonderful*) *modifications of instincts*, *sort of shield* (worker ants), *collection of fossil bones*, *relationship* (*between the dead and the living*), or *endless forms*. These expressions confirm that aspects such as excellence in the development of species or in their instinctive ability to adapt to the environment, including the complexity of the mechanisms that allow the human eye to work, are the type of biological manifestations that cause the greatest emotional agitation in Darwin. Similarly, Darwin applies the adverb *wonderfully* to emphasize the structural, functional and physical excellence of nature with expressions like *wonderfully perfect structure* (hive-bee’s), *wonderfully complex jaws and legs in crustaceans* and *wonderfully perfect* (prehensile organ).

In an attitude similar to that shown by the adjective *beautiful*, Darwin incorporates, from the fourth edition on, a vast number of expressions affected by the adjective *wonderful*, such as *differing manner* (offspring of two sexes), *the most wonderful of all cases* (alternate generations of animals) (4th ed.), *difference between worker ants and perfect females*, *thickness* (sedimentary strata), *changes of structure*, *law of the long endurance of allied forms*, *fact*, *organ* (the eye), *powers of the human eye*, *changes in function*, *one of the most wonderful animals in the world* (Greenland whale), *manner* (changing natural species), *co-adaptations*, *connecting link* (*Typotherium*), *case/s* (2 times), or *manner in which certain butterflies imitate other species*. It is noteworthy that a significant number of these additions are related to changes or differences in the structure and functions of living beings: *differing manner* (offspring of two sexes), *difference between worker ants and perfect females*, *changes of structure* or *changes in function and manner* (changing natural species).^18^

This lexical feature is similarly perceptible in the nouns affected by the adjective *prodigious*, predominating those cases where Darwin reports on the changing characteristics of his object of study, such as *geographical revolutions* and *transformations*, as well as expressions such as *amount of difference* (2 times) and *difference* (between ants) that express the differences he has found in a comparative study.

The updating of information—perceptible in the lexical evolution of the adjective *wonderful* and the adverb *wonderfully*—regarding the comparative observations Darwin makes between species, as well as his fascination—as seen in the lexical behaviour of the adjective *prodigious*—with the vast magnitude of geographical revolutions or the considerable difference that could exist between two types of ants, are specific evidences showing, first, that Darwin modifies the content of OS across editions based on the new results he witnesses, and, second, that the study of nature seems to be the path that allows Darwin to access a deeper dimension of understanding of the evolutionary mechanisms of living beings, as well as to qualify, with increasing enthusiasm,^19^ the impression they generate in him.

The feeling of amazement finds its most emphatic expression also in the use of the adjective *astonishing*, which Darwin applies to natural characteristics and facts such as *diversity of the breeds*, *improvement in many florists’ flowers*, *distance*, *power of diving*, *number of experiments*, *fact*/*s*, *rapidity*, *waste of pollen*, *number of species* and *result*. These lexical results are of a markedly naturalistic type, which make us infer that, again, the scientific study of nature is the primary basis without which Darwin would not be able to feel amazement at the natural events described. This is equally manifested in the lexicon affected by the adjective *marvellous*; Darwin does not marvel at visual aspects of the natural landscape, but with the *amount of diversification*, the *instinct/s*, the *fact*, the *characters*, the *case of Cecidomyia*, or the *manner* in which the Galapagos Islands are inhabited by very closely related species, that is, with characteristics of nature not noticeable if not from the perspective of scientific study.

In sum, the precise study of nature carried out over the years is fundamental to the updating of the results contained in OS. This is precisely the source of inspiration that generates in Darwin intense and growing feelings of beauty, wonder and astonishment.

#### Less frequent aesthetic-emotional adjectives and adverbs in OS

The semantic study of less frequent aesthetic-emotional adjectives and adverbs indicates that, despite their lower frequency in the OS texts, they have the characteristic of being the most markedly aesthetic—sometimes even poetic—and emotional ones. However, their application is almost exclusively restricted to markedly scientific-technical aspects of nature. These two lexical features, that is, a greater aesthetic-emotional nature, although less frequent, and the application strictly reserved for the scientific, mathematical and technical aspects of nature, are precisely attributes of disenchantment that acquire special visibility if the behaviour of the Darwinian lexicon is analysed across the six editions of OS.

As an example, the possible emotional use that could be applied to an adjective such as *delicate*, is ruled out if the nouns affected by this adjective are analysed. Darwin has rigorously limited its use to the description of forms, beings and properties of nature that have the quality of being delicate, such as *shells*, *hexagonal walls*, *nature* (quality), *cell-constructing work* (3rd ed.–5th ed.), *branching coralline*, *inhabitants of the cells*, *filaments*, *membrane*, *texture*, *inner coat of the eye* and *fleshy organs*. Many of the expressions just listed are introduced in the sixth edition of OS, with a striking difference of eight occurrences between the first and sixth edition. This, again, demonstrates Darwin’s progressive acquisition of scientific knowledge about nature and a consequent refinement of his descriptions.

However, unlike *delicate*, the adjective *exquisite* and the adverb *exquisitely* have been conscientiously applied in OS to aesthetic-emotionally denotate structural and adaptational features of nature, such as *exquisite adaptations*, *exquisite structure of a comb*, *exquisitely constructed hooks*, *exquisitely adapted parts and organs* and *exquisitely feathered gills*. These results indicate that the emotional intensity of the terms *exquisite* and *exquisitely* rests on the adaptive and structural excellence of natural objects, not admirable without the perspective of scientific optics. Darwin himself reaffirms his admiration for one of the examples just mentioned, the *structure of a comb*, indicating that he perceives it with *enthusiastic admiration*.^20^

As far as admiration is concerned, it should be noted that Darwin uses *admirably* four times also to refer to the excellence of the adaptation to the environment of some living beings: *admirably adapted woodpecker*, *admirably adapted pleuronectidae*, *admirably adapted pollinium* and *admirably adapted species*. In other words, Darwin’s lexicon shows, once more, that his admiration lies with the complexity and functional and adaptive excellence of biological objects and beings.

The list of lexical examples that manifest a union between aesthetic-emotional adjectives and adverbs, and natural objects, states, attributes and processes, some of marked scientific-technical characteristics, expands, in this respect, considerably. The surprising emphasis transmitted by the adverb *astonishingly* affects only technical aspects of nature such as artificially improved varieties (*astonishingly improved breeds by crossing them*) and the rapid increase of some animal species (*astonishingly rapid increase of various animals*); in a similarly emphatic, although also scientific-technical, way, Darwin adjectivizes the sea as a *formidable barrier* that might interfere with the geographical distribution of animal species; the adjective *magnificent*, of equivalent expressive intensity, applies exclusively to the *compound eyes* of butterflies in a state of chrysalis; the adverb *marvellously* is used to indicate the perfection of the attributes of the eye (*marvellously perfect attributes*/*characters*); the adverb *nicely* accentuates the perfect relationship of balance between, on the one hand, variability of the forces of competing organic beings (*nicely balanced forces*) and, on the other hand, the consequent fluctuating stability of the scale of victory and defeat in the struggle for life (*nicely balanced scale in the struggle for life*); the adjective *stupendous* affects only the noun *degradation*, referring to the deterioration of some volcanic islands; and finally, the adjective *wondrous* refers to the electrical organs of some fish (*wondrous organs*) and to the beautiful physical organisation of some living beings classified as low on the scale of nature (*really wondrous and beautiful organisation*).

These examples prove that although Darwin does not cease to experience intense emotions of aesthetic magnificence, admiration and surprise before the excellence of nature, the use of these aesthetic-emotional adjectives and adverbs is mostly restricted to scientific-technical aspects of nature. However, this restriction is not absolute. Without diverting attention from his primary purpose in OS, that is, to explain the natural mechanisms necessary for a correct dissemination of evolutionary ideas, Darwin occasionally describes nature with a characteristically aesthetic-poetic lexicon, referring both to merely aesthetic aspects of the landscape and natural objects, as well as to his mood. Thus, for example, the way Darwin emphasizes the delicacy by which bees spread the vermilion colour of the wax through the axes of the hive cells is especially striking: *as delicately as a painter could have done with his brush.* Similarly, Darwin also describes some *birds* and the *plumage* of birds of paradise as *gorgeous*, certain fruit varieties as *splendid*, the *beauty in scenery* as *picturesque*, the *diversity of nature* as *harmonious* and butterflies as *magnificently coloured.*

In sum, although these expressions, when analysed individually, are those with the highest aesthetic-poetic intensity compared to the most frequent adjectives and adverbs analysed in sub-chapter 3.1.1., it should be noted that, if analysed in the textual context of OS, they all are inserted in paragraphs rigorously dedicated to the scientific explanation of natural facts and mechanisms. In addition, the presence of these adjectives and adverbs in the text is minimal, as they appear, except for the adjective *delicate*, once or twice in each edition of the work, and in the case of the adjective *picturesque*, only once in the fifth edition.

### Semantic analysis of religious or spiritual lexicon in OS

The irregular lexical behaviour of religious or spiritual adjectives, in terms of terminological additions and subtractions is referred to, is visually striking when compared with the generalized semi-stable or growing lexical tendency of aesthetic-emotional adjectives. Nevertheless, this irregularity depends, to a large extent, on the additions or deletions that Darwin applies to the OS bibliographic material, where the majority of these adjectives are included. Religious or spiritual adjectives, therefore, have no theoretical weight in any of the six editions of OS.^21^ OS is, consequently, a disenchanted text, in the strictest sense of the term, that is, lacking references to supra-natural entities that are theoretically relevant to the scientific argumentation of OS.

The adjective *divine* shows, precisely, a markedly irregular lexical behaviour. It affects the nouns *power* and *love* in the first edition and *author* in the second edition; in the third edition Darwin eliminates the noun *love* on the two occasions in which it appears; in the fourth edition he adds the noun *elements*; finally, the fifth and sixth editions maintain the number of occurrences of the third edition. These lexical results, however, lack theoretical value for the OS text. The expression *divine power* belongs to Whewell, which Darwin quotes;^22^ the expressions *divine love* and *divine elements* are found in the bibliographic content of OS; finally, the expression *divine author* obviously refers to an author mentioned by Darwin.

The adjective *holy* behaves similarly in OS. It affects the nouns *land*, *altar* and *places*, which are included in the first and second editions, eliminated in the third, retaken up in the fourth edition, with the addition of the noun *scripture*, and finally eliminated in the fifth and sixth editions. Now, all these expressions belong to the bibliographic list of OS, that is, to the works of other authors. The semantic value of the adjective *holy*, as well as of all the nouns affected, is, therefore, argumentatively null for the content of OS. Likewise, the only occurrence of the adjective *mystical*, from the third to the sixth edition of OS, affects the noun *natur-philosophie*, but it is a reference by Darwin to Oken’s work. A case analogous to those of the adjectives *holy* and *mystical* is that of the adjective *sacred*, whose only two occurrences are *beetle of the Egyptians*, a type of beetle also called *Ateuchus*, and *places*, which belongs to the bibliography. We find the same lexical situation in the case of the adjective *supernatural,* which is used to quote Butler^23^ from the second edition on (*what is supernatural or miraculous…*), and to refer to part of the content of Guizot’s work (*The Supernatural*) in the fourth edition.

*Immaterial* and *mysterious* are not even used in OS as adjectives of a religious or spiritual type, but, in the case of *immaterial*, as a synonym for “irrelevant”; in the case of *mysterious*, as a synonym for “unknown”, as it refers to aspects of Darwin’s investigations that are unknown, or that have ceased to be unknown to him, like *laws of the correlation of growth*, *causes*, *the succession of the same types of structure*, *a manner* and *cases of correlation*.

Lastly, *miraculous* is the only adjective which has minimal, and indirect, theoretical value in OS, as it affects the expression *act/s of creation* and the nouns *interposition* and *process*, included in the text as examples of miraculous cases of creation, interposition and process incompatible with Darwin’s theory of evolution based on natural selection. The only utility Darwin obtains from the use of the adjective *miraculous* is, therefore, to complete the scientific content of OS with examples of theoretical incompatibility.

In brief, as advanced above, since Darwin does not use any of the religious or spiritual adjectives as argumentative foundation for his explanations in OS, it is therefore possible to conclude that disenchantment also manifests itself in the texts in the form of the absence of religious or spiritual adjectives with relevant theoretical value.

## What about Darwin’s “colour-blindness”? An explanatory hypothesis on its source^24^

The lexically strict, although emotively optimistic, manifestation of disenchantment in Darwin’s six editions of OS that has been depicted so far, however, seems to be in conflict with some statements that Darwin includes in his *Autobiography* (AB) about a supposed, and unwanted, loss of the capabilities of aesthetic and artistic perception, as anticipated in the introduction of this paper. The AB extract where Darwin confesses to experience colour blindness (Barlow 2005, p. 76) is especially significant in that it contrasts the positively disenchanted view of nature that Darwin projects not only in the last OS paragraph, but throughout the six editions of the work, as demonstrated in the semantic analysis of the Darwinian lexicon. To Darwin’s astonishment, the magnificent scenes of nature that led him to believe that there is more in man than the breath of his body^25^ have ceased to provoke the same feelings in him with the passage of time. Could these symptoms of sentimental blindness that Darwin self-diagnoses be related to a parallel loss of artistic tastes?^26^ This chapter will delve into the confidences that Darwin exposes in AB and will propose, as a complementary hypothesis that refines the conclusions reached so far, a solution to the alleged problem of Darwinian disenchantment.

Darwin notices a change in his thoughts (“my mind has changed over the last twenty or thirty years” (Barlow 2005, p. 76)) that manifests itself, on the one hand, in an unwanted perception of poetry as boring and nauseating, in the case of Shakespeare (“I have tried lately to read Shakespeare, and found it so intolerably dull that it nauseated me” (Barlow 2005, p. 113)), and, on the other hand, in the loss of taste for pictures or music (“I have also lost my taste for pictures or music” (Barlow 2005, p. 113)). Darwin claims to maintain a certain taste for refined natural settings, although he admits to have lost the feeling of absolute enjoyment that such scenes used to cause him years earlier (“I retain some taste for fine scenery, but it does not cause me the exquisite delight which formerly did” (Barlow 2005, p. 113)). These symptoms are synthesized in what Darwin, regretfully, diagnoses as a strange loss of the most refined aesthetic tastes. However, Darwin confirms to maintain interest in readings focused on history, biographical accounts, travel literature and essays of all kinds (“This curious and lamentable loss of the higher aesthetic tastes is all the odder, as books on history, biographies, and travels (independently of any scientific facts which they may contain), and essays on all sorts of subjects interest me as much as they did” (Barlow 2005, p. 113)). An intense feeling of confusion and a need to find answers emerge from this retrospective analysis in search of the mental changes that Darwin has experienced over the years.

The allusion, in brackets, to the possibility of finding a scientific background in the readings on which he still can stand to focus (“independently of any scientific facts which they may contain”), must not go unnoticed. The scientific content for which Darwin has not experienced disinterest becomes the main cause on which Darwin projects his suspicions about a possible partial cerebral atrophy (“why this should have caused the atrophy of that part of the brain alone, on which the higher tastes depend, I cannot conceive” (Barlow 2005, p. 113)). Darwin’s mental transformation would consist of a hypothetical mechanization of the mind, which has become, according to his own harsh words, “a kind of machine for grinding general laws out of large collections of facts” (Barlow 2005, p. 113). Cerebral palsy linked to the highest tastes, apparently produced by an over-dedication to scientific study, remains an incomprehensible key, inducer of such a pronounced confusion that leads Darwin to elucidate over possible methods that would have prevented the state of perceptual and emotional decline described, such as a more regular approach to music or poetry (“if I had to live my life again, I would have made a rule to read some poetry and listen to some music at least once every week; for perhaps the parts of my brain now atrophied would thus have been kept active through use” (Barlow 2005, p. 113)).

Let us accept for a moment the hypothesis that contemplates an over-concentration in scientific study as the main cause of Darwin’s supposed cerebral atrophy. What the acceptance of this specific interpretation does not contemplate is the author’s logical bewilderment in regard to the explanatory implications that such an admission would entail. If scientific over-dedication is assumed as the cause of Darwin’s atrophy, then the reason why such scientific practice is exclusively harmful to higher aesthetic tastes and not to appreciation for and delight with historical, biographical or literary subjects should be argued accordingly. This particular reading is resolutely as inefficient as Darwin’s vague attempts to consider the regulated follow-up of musical habits and poetic reading as a precautionary method of avoiding all kinds of mental atrophy due to disuse.

The hypothesis that perhaps best accounts for the origin of the newly noted weakening of Darwin’s emotional side, is that which focuses on the modification of his religious beliefs. In AB, Darwin recalls his absolute conviction of the existence of God and the immortality of the soul citing a passage from the *Journal of Researches* (1860a) where he highlights the emergence of feelings of astonishment, admiration and devotion in the Brazilian jungle of such a pronounced vehemence, that would evade any tight description: “In my Journal I wrote that whilst standing in the midst of the grandeur of a Brazilian forest, ‘it is not possible to give an adequate idea of the higher feelings of wonder, admiration, and devotion, which fill and elevate the mind’” (Barlow 2005, p. 76). The expressive inability in the face of the sublime grandeur^27^ of nature, would be intimately linked to the feeling that “there is more in man than the mere breath of his body” (Darwin 1860a, p. 503). However, the colour blindness that Darwin claims to suffer, would have blocked the process of aesthetic perception of nature and the subsequent emergence of religious feelings.

The experiences reported in the *Journal of Researches* would be supported by the classic link between the feeling of the sublime and the religious feeling, support that would crumble over the years with the progressive evolution of Darwin’s thinking. Darwin himself confirms this clearly: “The state of mind which grand scenes formerly excited in me, and which was intimately connected with a belief in God, did not essentially differ from that which is often called the sense of sublimity” (Barlow 2005, p. 76). ¿Could Darwin have been nostalgic for Paleyan natural theology, that is, the perception of nature as *vestigia Dei*?

In *Natural Theology* (1802), William Paley attributes, by inductive inference, the evidences of design in nature like the human eye, which Paley compares with a telescope, to the existence of a divine designer; such a complex natural mechanism like that of the human eye must have had a designer, just as the machinery of a telescope had. Paley’s work was of considerable importance to Darwin; the argument from design in nature resulted, initially, in being absolutely convincing until natural selection was discovered.^28^ Darwin himself states this in AB: “The old argument of design in nature, as given by Paley, which formerly seemed to me conclusive, fails, now that the law of natural selection has been discovered. We can no longer argue that, for instance, the beautiful hinge of a bivalve shell must have been made by an intelligent being, like the hinge of a door by man” (Barlow 2005, p. 73). Darwin belongs to the generation that participates in the old “onto-theological”—in the Heideggerian sense—paradigm. In this paradigm reigned a non-disenchanted worldview that considered beauty in nature, like the hinge of a bivalve shell, especially when it is understood as a manifestation of the sublime, as a vestige of divinity. However, the hypothesis of natural selection brought with it the establishment of a new paradigm, naturalized, naturalistic and disenchanted.

So, if the supernatural turns out to be the only resource that can account for what has been experienced in the face of majestic scenes of nature capable of generating a feeling of sublimity, and Darwin alludes to an irremediable supposed loss of faith over time, it is understandable that he claimes to suffer a type of colour blindness, a defective perception before the sublime scenarios of nature. It is, however, striking that Darwin did not later clarify that what is truly defective, that is, the cause of the sensation of colour blindness, is the missing religious link of the triad 1. Perception of the beauty of nature; 2. Religious feeling; 3. Experimentation of the sublime, and not the ability to perceive beauty.

I therefore deduce the existence of two types of perceptual losses—that are not opposed, but overlapping—that will allow us to devise a solution to Darwin’s problem. First, the loss referring to cerebral atrophy, erroneously, in my view, attributed to an over-dedication to scientific activity. Second, the loss produced by disenchantment, in an emotionally negative sense, with respect to the landscape aesthetic perception, that is, a supposed colour blindness derived from the loss of religious beliefs; this loss of aesthetic perception caused by disenchantment, should, nevertheless, have been specified as a *modification* of perception, and not as a loss.

The error of Darwin’s interpretation could be of a syllogistic nature. His mind seems to operate as follows: (a) There is aesthetic experience if—in nature or art—the contents XYZ are perceived; (b) I do not perceive them anymore; (c) Then, I no longer have aesthetic sensibility. However, premise (a) is arbitrary, something that Darwin, due to cultural or personal reasons, perhaps, could not notice.

This syllogistic deduction could have led Darwin to believe that, similarly, he has lost the taste for the higher aesthetic tastes. If Darwin identifies the sublime with transcendence, with the imprint of divinity in nature, and he ceases to establish such a relationship, given the ideological demands consequence of the assimilation of his theory of evolution, it is therefore plausible to accept that he believed to have lost, at the same time, the feeling for the higher aesthetic tastes—linked to arts which could equally have led Darwin to experience feelings of religious exaltation—and for certain aspects of the natural beauty that he could initially perceive. It is then possible to believe that both feelings of loss have a common root: the profound change that his scientific theory causes to his worldview, namely, the alteration and destruction of many of the basic assumptions of the pre-Darwinian worldview.

In sum, not only the colour blindness passage, but also that of cerebral atrophy could be related to the loss of perception of the supernatural in nature. This could have caused Darwin the feeling of having lost aesthetic sensibility.

## Conclusions

Firstly, the question of whether disenchantment can be compatible with aesthetic experience and sensibility to beauty must be taken up again. The results obtained from the semantic analysis of the lexical variations and frequencies across the six editions of OS have shown that the disenchantment of the world —in the way in which it has been perceived culturally and historiographically as a desacralized conception of the world—, on the one hand, clearly manifests itself in the texts with lexical facts such as a semantically irrelevant presence of religious or spiritual lexicon and, on the other hand, is compatible, regardless of what Darwin occasionally affirmed in AB, with aesthetic sensibility to natural beauty; the results of the semantic analysis, such as the use of aesthetic-emotional adjectives and adverbs applied to nature’s structurally constitutive characteristics, indeed show that it is not only compatible, but can further refine that sensibility.

These natural characteristics would not be noticeable, and therefore, not aesthetically qualifiable, without the influence of a scientific-technical knowledge base on nature. Unlike what could be assumed by adopting a pessimistic perspective on the concept of disenchantment, the lexical content of OS demonstrates that Darwinian science, despite bringing to light the biological structure of the multiplicity of natural facts, instincts, mechanisms, etc., provokes in Darwin intense aesthetic-emotional feelings precisely focused on the elegance and functional beauty of nature that he himself has explained.

In sum, as can be inferred from the semantic analysis of the lexical results, despite Darwin’s conception of the world is supported by disenchanted ontological pillars, his view of nature has not been aesthetically weakened.

Secondly, in regard to the question about whether it was the disenchanted conception of the world that led Darwin to believe himself colour blind in the face of natural beauty, it is possible to confirm that the type of disenchantment Darwin describes in AB, that is, the cessation of religious belief, apparently brings him closer to the concept of disenchantment, suggested by Weber, that has a negative connotation: “atrophy” and “colour-blindness” are not terms compatible with a complete, intense and positively disenchanted aesthetic perception of the landscape, or more specifically, with the aesthetically and scientifically inspiring view of the world proposed by Darwin, as evoked in the concluding lines of OS.

However, it is not possible to infer this negatively disenchanted view from the study of the lexical evolution of the different editions of OS, because, as has been demonstrated, the variations of the Darwinian lexicon show a growing fascination, above all, for constitutive, essential aspects of nature. So, we could state, at least tentatively, that Darwin could perhaps have confused his feelings of an inexplicable lack of interest in landscape aesthetics and the arts. Given the correlation between perception, in nature, of beauty, of the sublime in it, and of the feeling of the supernatural, inferred through the commemoration of the passage from the *Journal of Researches* which reveals the conviction that there is something more in man than the mere breath of his body, the loss of religious feeling, in conjunction with the detection of an atrophy with respect to the finest arts, could have driven Darwin to deduce, perhaps wrongly, a loss of the ability in aesthetic perception in general, instead of considering it to be a modification of perception.

In short, the cause of Darwin’s error of interpretation of his aesthetic sensibility would then have been to assume the equation of equality between aesthetic sensibility in the face of nature and the perception of its beauty as part of the *vestigia Dei.*

## Supplementary Information

Below is the link to the electronic supplementary material.

Supplementary information 1 (PDF 422 kb)

## Footnotes

1 In fact, Darwin may have been inspired by a similar paragraph in A. von Humboldt’s *Personal Narrative* (Wulf 2015, p. 234). Among the expert interpretations of the last lines of OS, I highlight: Cannon 1968; Kohn 1997; Levine 2008.

2 Darwin added the specifying expression “by the Creator” after “breathed” in the second edition of OS (1860b, p. 484), an addition “that he later regretted” (Browne 2002, p. 96).

3 Here is a selection of excellent research pieces on the origin of OS: Vorzimmer 1975; Schweber 1977; Kohn et al. 1982.

4 Although Darwin’s autobiography was published, along with part of his correspondence, in three volumes in 1887 under the edition by his son Francis Darwin with the title *The Life and Letters of Charles Darwin, Including an Autobiographical Chapter*, I will quote the edition by N. Barlow: *The Autobiography of Charles Darwin, 1809–1882* (2005).

5 Among the extensive bibliography on the concept of “secularization”, I highlight *A Secular Age* (2007) by C. Taylor, where he presents the evolution of the process of the secularization of the world throughout history.

6 Although the concept “disenchantment of the world” is generally attributed to Max Weber, the poet Friedrich von Schiller (1962) had previously alluded to the “dis-godding of nature” (*die Entgötterung der Natur*) in his 1788 25-stanza-poem “The Gods of Greece” (*Die Götter Griechenlandes*), where he refers to the passing of an age in which divinity inhabits the world to a godless world, and thus, a godless nature. But even before Schiller, the philosopher René Descartes, in the seventh chapter of his 1664 work *Le Monde de R. Descartes ou le Traité de la Lumiére*, describes nature not as a Goddess but as matter itself with its qualities: “Sachez donc, que par la Nature je n’entends point ici quelque Dèesse, ou quelque autre sorte de puissance imaginaire, mais que je me sers de ce mot pour signifier la Matière même en tant que je la considère avec toutes les qualités que je lui ai attribuées” (Adam and Tannery 1974, p. 37).

7 Adorno and Horkheimer (2016) detected the weight of the disenchanting program in the Renaissance, focused on “the dissolution of myths and the substitution of knowledge for fancy” (p. 3). The disenchanted Renaissance nature got rid of animism, gods, myth and superstition; everything that was not subject to numerical, computational rules, become illusory and suspicious. The Renaissance human used and dominated disenchanted nature —a demystified nature, subject to the rules of calculability and reduced to mere objectivity— for his own interests. Adorno and Horkheimer’s criticism was therefore directed at the progressively dehumanizing Renaissance reality that fostered reason and progress.

8 On the romantic foundations of Darwin’s theory proposed by Richards see: Richards 2002; Richards and Ruse 2016.

9 Among the literature that manifestly defends that scientific knowledge of nature allows us to observe it from an aesthetically reinforced perspective, and not vice versa, I highlight Pearson 1892; Beer 1983; Dawkins 1998; Fisher 1998; Carlson 2000; Parsons 2008; Campion 2011.

10 The *Online Variorum of Darwin’s Origin of Species*, created by Barbara Bordalejo (2012), is available at http://darwin-online.org.uk.

11 Although recent studies (Mahmoudi and Abbasalizadeh 2019) have argued in favour of the enriching approach that statistics and text mining strategies can offer to literary studies, other pieces of research (Da 2019) warn us about the problems, logical fallacies and conceptual flaws that could arise in computational literary studies.

12 An example of trans-natural semantics would be to make use of the notion of *beauty* referred to nature assuming that its meaning necessarily refers to the religious ontological domain. But the fact that this use has been historiographically very frequent does not demonstrate that it is logically necessary.

13 See, for instance, George Campbell’s, eighth Duke of Argyll, discussion on beauty in his work *Reign of Law* (1867), an attack on the evolutionary theories of Darwin.

14 Please see Table 1 included in the Electronic Supplementary Material separate PDF file.

15 Please see Graph 1 included in the Electronic Supplementary Material separate PDF file.

16 Please see Table 2 included in the Electronic Supplementary Material separate PDF file.

17 All the adjectives, adverbs and expressions affected by them, included in Table 2 (Online Resource, pp. 4–11), are of a high semantic relevance. Nevertheless, although it would be desirable to comment about all the lexical minutiae discovered in the semantic analysis of the Darwinian lexicon, which have in fact favourably contributed to formulating these conclusions, I will analyse a reduced amount of the lexical results for reasons of spatial economy.

18 In this respect, it is important to note that the adjective *wonderful* is occasionally used to express a type of descriptive emphasis referring to a fact of marked specific characteristics, striking rarity or oddity, and not only as a merely emotional qualifier. Concrete cases that exemplify this aspect of Darwin’s language are the newly highlighted expressions about changes or differences detected in physical, structural, functional or behavioural aspects of some living beings. However, despite the remarkable naturalistic nature of these expressions, they entail a considerable mood connotation. The use of the adjective *wonderful*, and not another that specifies the type or degree of change or difference that Darwin refers to in these expressions in a more precise and technical way, serves to visualise a background of intense emotional and intellectual interest. This warning could be equally applicable to the adjective *extraordinary* and the adverb *extraordinarily*, for, although they mostly affect nouns referring to elements and attributes of nature that are exceptional or unusual, the textual context in which these terms are immersed usually describes Darwin’s intellectual enthusiasm.

19 Darwin’s growing enthusiasm can be measured, precisely, by the evolution of occurrences of the adjective *wonderful* throughout OS editions. While in the first three editions the number of occurrences remains at 27, in the fourth edition it increases by two (29), in the fifth edition it increases by four in comparison to the fourth edition (33) and in the sixth edition it increases by eight in comparison to the fifth edition (41). If we compare the number of occurrences of the adjective *wonderful* in the first and last editions of OS, there is an increase of 14 cases.

20 See the adjective *enthusiastic* in Table 2 (Online Resource, pp. 4–11).

21 Nevertheless, this idea is not in conflict with views claiming that the use of theology played an epistemic role in the OS’s case for evolution, and against special creation (Dilley 2012). Excellent analyses on Darwin’s use of theology can also be found in Gillespie 1979; Ospovat 1980; Brooke 1985, 2008; Kohn 1989; Depew 2008, to mention a few.

22 However, as Topham (2010, p. 111) advocates, this quotation, belonging to W. Whewell’s *Bridgewater Treatise*, and located “in a prominent position opposite the title page of the *Origin of Species*”, was in support of the theology of natural laws, despite Darwin’s “subsequent erosion of his belief in God into a somewhat vacillating form of agnosticism”. It goes without saying, Topham notes, that the “nature of Darwin’s quotation” is “strategic”.

23 This quotation was added as a nod to the theological concerns of some of Darwin’s acquaintances (Browne 2002, p. 96).

24 A more extensive version of this chapter is included in my doctoral thesis (Jiménez Pazos 2016).

25 Darwin’s memories of youth having resulted in a supposed perceptual colour blindness in his old age are included in *Journal of Researches*: “Among the scenes which are deeply impressed on my mind, none exceed in sublimity the primeval forests undefaced by the hand of man; […] —no one can stand in these solitudes unmoved, and not feel that there is more in man than the mere breath of his body” (Darwin 1860a, p. 503).

26 There are several authors who have contributed to the explanation of Darwin’s loss of aesthetic taste. See, for example: Campbell 1974; Fleming 1961; von Sydow 2005.

27 On the concept of the sublime in Darwin’s works, I recommend the following research works: Bradley 2011; Levine 2011; Larson 2013.

28 The Paley-Darwin confrontation is a too simplistic one. In the middle of both are the *Bridgewater Treatises*, a series of eight works, written by Paley’s successors, that proposed alternative approaches to nature in the frame of natural theology. Darwin owes, according to Topham (2010), “a debt to the *Bridgewater Treatises*” (p. 112) for they trod “the path between Paley’s watchmaker and Darwin’s natural selection” (p. 89) […] [b]y adapting natural theology to a progressive and law-like view of the history of creation” (p. 112). For a complete understanding of the dimension that the debates around the question of purpose in nature acquired from the XVII^th^ Century on, as well as Darwin’s intellectual involvement in these debates, I highlight Ospovat’s *The Development of Darwin’s Theory* (1980).
