# Correction to: Darwin’s perception of nature and the question of disenchantment: a semantic analysis across the six editions of *On the Origin of Species*

**DOI:** 10.1007/s40656-021-00437-z

**Published:** 2021-06-07

**Authors:** Bárbara Jiménez-Pazos

**Affiliations:** 1 IAS-Research - Centre for Life, Mind and Society, Philosophy Department, University of the Basque Country, San Sebastián, Spain; 2 Facultad de Educación, Filosofía Y Antropología, Universidad del País Vasco, Avenida de Tolosa 70, 20018 San Sebastián, Guipúzcoa Spain

## Correction to: HPLS (2021) 43:57 https://doi.org/10.1007/s40656-021-00373-y

Footnotes 24 and 25 in the online version of above mentioned article do not correspond to footnotes 24 and 25 in the downloadable version of the paper (PDF). The correct footnotes 24 and 25 are published in the PDF and read:

^24^A more extensive version of this chapter is included in my doctoral thesis (Jiménez Pazos 2016).

^25^Darwin’s memories of youth having resulted in a supposed perceptual colour blindness in his old age are included in Journal of Researches: “Among the scenes which are deeply impressed on my mind, none exceed in sublimity the primeval forests undefaced by the hand of man; […]—no one can stand in these solitudes unmoved, and not feel that there is more in man than the mere breath of his body” (Darwin 1860a, p. 503).

The original article has been corrected.

