# Supplementary material for: Darwin’s perception of nature and the question of disenchantment: a semantic analysis across the six editions of On the Origin of Species
Source: Hist Philos Life Sci. 2021 Apr 14;43(2):57. doi: 10.1007/s40656-021-00373-y (PMC8046690; doi:10.1007/s40656-021-00373-y)
Supplement: Supplementary file 1 — Supplementary information 1 (PDF 422 kb) [file 40656_2021_373_MOESM1_ESM.pdf]

ELECTRONIC SUPPLEMENTARY MATERIAL

Darwin's perception of nature and the question of disenchantment: a semantic analysis across the six editions of *On the Origin of Species*

History and Philosophy of the Life Sciences

Bárbara Jiménez-Pazos

barbara.jimenez@ehu.eus

IAS-Research. Centre for Life, Mind and Society. Philosophy Department, University of the Basque Country, San Sebastián, Spain.

Facultad de Educación, Filosofía y Antropología, Universidad del País Vasco, Avenida de Tolosa 70, 20018 San Sebastián, Guipúzcoa, Spain.

Table 1: Total count of the occurrences of aesthetic-emotional and religious or mystical adjectives and adverbs in the six editions of the *Origin*

**Adjectives**	**Occurrences**					
	**1st ed.**	**2nd ed.**	**3rd ed.**	**4th ed.**	**5th ed.**	**6th ed.**
Admirable	19	19	20	20	21	23
Admirably	2	2	2	3	2	6
Astonishing	8	8	9	11	12	12
Astonishingly	2	2	2	2	2	2
Attractive	6	6	6	6	4	4
Beautiful	15	15	19	34	34	35
Beautifully	5	5	6	7	7	9
Delicate	2	2	4	5	5	10
Delicately	2	2	2	2	2	2
**Divine**	**3**	**4**	**2**	**5**	**2**	**2**
Enthusiastic	1	1	1	1	1	1
Exquisite	2	2	2	2	2	2
Exquisitely	2	2	2	2	3	3
Extraordinary	21	22	23	24	27	32
Extraordinarily	12	11	12	13	12	12
Formidable	0	0	0	0	2	3
Gorgeous	1	1	1	2	2	2
Harmonious	1	1	1	2	2	1
Harmoniously	0	0	0	1	1	1
**Holy**	**5**	**5**	**0**	**9**	**0**	**0**
**Immaterial**	**2**	**2**	**3**	**3**	**4**	**4**
Magnificent	1	1	1	1	1	1
Magnificently	0	0	0	1	1	1
Marvellous	5	5	5	5	5	6
Marvellously	0	0	0	1	1	0
**Miraculous**	**2**	**3**	**5**	**5**	**5**	**5**
**Mysterious**	**4**	**4**	**4**	**4**	**4**	**5**
**Mystical**	**0**	**0**	**1**	**1**	**1**	**1**
Nicely	3	3	3	3	3	3
Picturesque	0	0	0	0	1	0
Pretty	3	3	3	3	2	2
Prodigious	9	9	9	9	7	9
**Sacred**	**1**	**1**	**1**	**2**	**1**	**1**
Splendid	2	2	2	2	2	2
Stupendous	1	1	1	1	1	1
**Supernatural**	**0**	**1**	**1**	**2**	**1**	**1**
Sweet	5	5	5	5	4	4
Wonderful	27	27	27	29	33	41
Wonderfully	7	7	6	8	8	16
Wondrous	1	1	2	2	2	2

Graph 1: Visual distribution of the occurrences of aesthetic-emotional and religious or mystical adjectives and adverbs in the six editions of the *Origin*

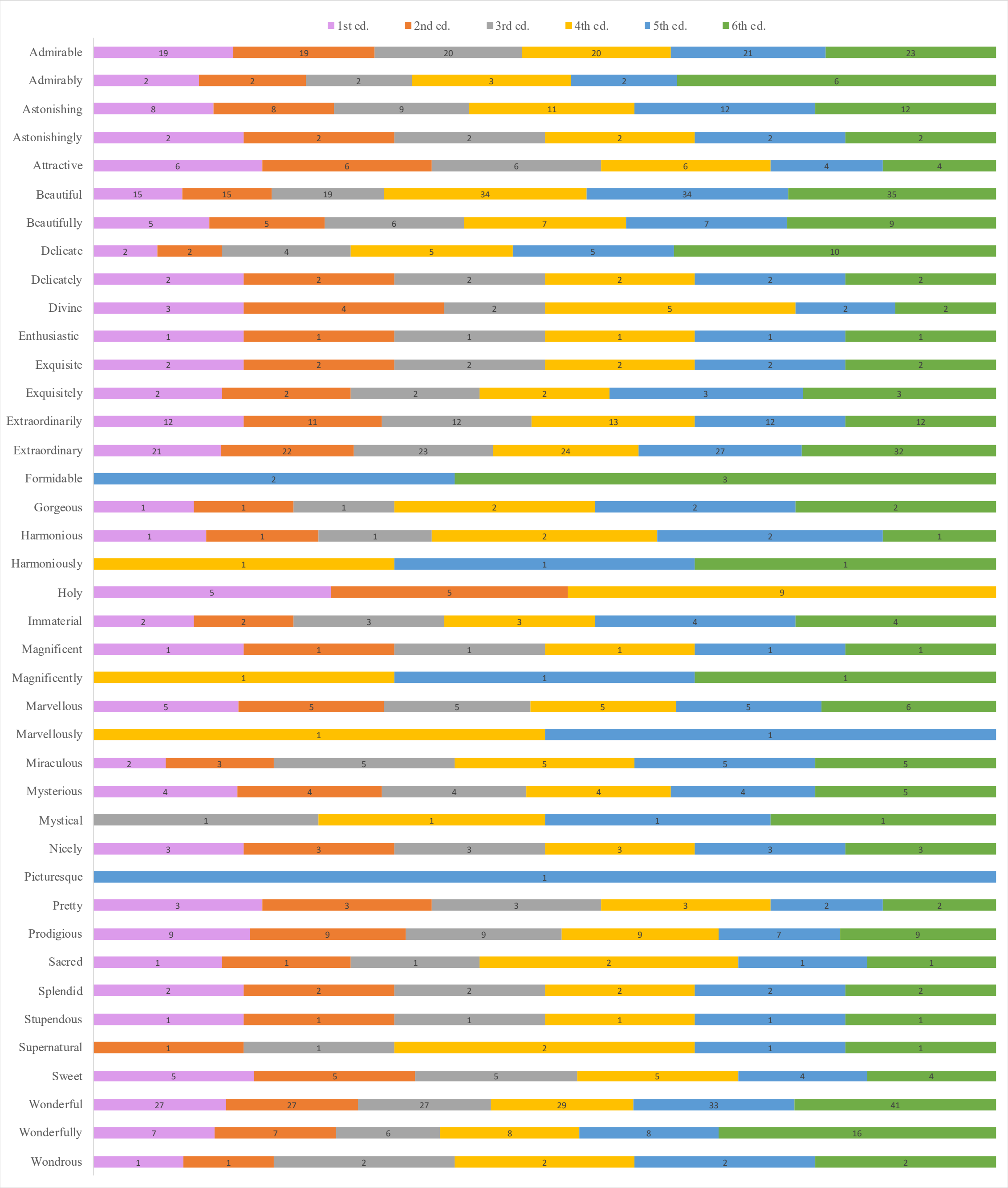

Table 2: Expressions affected by aesthetic-emotional and religious or mystical adjectives and adverbs in the six editions of the *Origin*

Adjectives and Adverbs	Affected expressions					
	**1st ed. 1859**	**2nd ed. 1860**	**3rd ed. 1861**	**4th ed. 1866**	**5th ed. 1869**	**6th ed. 1872**
ADMIRABLE	-VARIETIES (strawberry)<br>-WORK (4 times)<br>-DIVISION of labour (ants)<br>-OBSERVERS (2 times)<br>-LESSON (to stand on the North Downs…)<br>-PALAEONTOLOGIST (Edward Forbes)<br>-MEMOIRS (Mr. Prestwich)<br>-'INTRODUCTION to…' (Dr. Hooker)<br>-CLEARNESS (Dr. Hooker)<br>-ACCOUNT of Bermuda (Mr. J. M. Jones)<br>-ZEAL and researches of Mr. Wallace<br>-MEMOIR (Dr. Hooker)<br>-DRAWINGS by Prof. Huxley<br>-ARCHITECTURAL powers (hive-bee's)<br>-MANNER (tracing the former migrations thanks to geology)	*Same as 1st ed.*	*Same as 1st ed.*<br>–<br>~~-LESSON (to stand on the North Downs…)~~<br>+<br>-REVIEW (of the *Origin* by Asa Gray)<br>-FORCE and clearness (Mr. Wallace's promulgation of natural selection)	*Same as 3rd ed.*<br>–<br>~~-DIVISION of labour (ants)~~<br>~~-REVIEW (of the *Origin* by Asa Gray)~~<br>+<br>-PAPER (by Naudin)<br>-RESEARCHES (Prof. Rutimeyer)<br><br>-RESEARCHES of Mr. Wallace (see 1st ed.)<br>-FLORA (Dr. Hooker) (see 1st ed.)	*Same as 4th ed.*<br>+<br>-ESSAY (by Nageli)	*Same as 5th ed.*<br>–<br>~~-CLEARNESS (Dr. Hooker)~~<br>+<br>-MEMOIR (Hensen)<br>-ART and force (Mivart objections against natural selection)<br>-MEMOIRS (Mr. Croll)
OCCURRENCES	19	19	20	20	21	23
ADMIRABLY	-ADAPTED (woodpecker)<br>-DISCUSSED accounts by Alph. De Candolle	*Same as 1st ed.*	*Same as 1st ed.*	*Same as 1st ed.*<br>+<br>-WELL (Agassiz's accordance with natural selection)	*Same as 4th ed.*<br>–<br>~~-DISCUSSED accounts by Alph. De Candolle~~<br><br>-WELL (Agassiz's and others' accordance with Darwin's theory) (see 4th ed.)	*Same as 5th ed.*<br>+<br>-ADAPTED (Pleuronectidae)<br>-ADAPTED (a highly complex pollinium)<br>-ADAPTED (species)<br>-AGREE with the theory of descent with modification (leading facts in palaeontology)
OCCURRENCES	2	2	2	3	2	6
ASTONISHING	-DIVERSITY of the breeds<br>-IMPROVEMENT in many	*Same as 1st ed.*	*Same as 1st ed.*<br>+	*Same as 3rd ed.*<br>+	*Same as 4th ed.*<br>+	*Same as 5th ed.*

	florists' flowers<br>-DISTANCE from tree to tree<br>-POWER of diving<br>-NUMBER of experiments<br>-FACT<br>-RAPIDITY (spreading of species)<br>-WASTE of pollen		-NUMBER of species	-One of the most astonishing FACTS<br>-FACT (a delicate branching coralline producing a host of huge floating jelly-fishes)	-RESULT	
OCCURRENCES	8	8	9	11	12	12
ASTONISHINGLY	-IMPROVED breeds by crossing them<br>-RAPID increase of various animals	*Same as 1st ed.*	*Same as 1st ed.*	*Same as 1st ed.*	*Same as 1st ed.*	*Same as 1st ed.*
OCCURRENCES	2	2	2	2	2	2
ATTRACTIVE	-PARTNER<br>-PEACOCK (attractive to all his hen birds)<br>-Horn-like PROTUBERANCES (attractive to the females)<br>-FLOWERS<br>-PLANT (attractive to insects) (2 times)	*Same as 1st ed.*	*Same as 1st ed.*	*Same as 1st ed.*	*Same as 1st ed.*<br>–<br>~~-Horn-like PROTUBERANCES (attractive to the females)~~<br>~~-FLOWERS~~	*Same as 5th ed.*
OCCURRENCES	6	6	6	6	4	4
BEAUTIFUL	-BLUE colour (bird)<br>-RACES of plants<br>-CO-ADAPTATIONS<br>-ADAPTATION/S (2 times)<br>-DIVERSITY and proportion of kinds<br>-MALES<br>-Really beautiful and elaborate CONTRIVANCE<br>-RAMIFICATIONS<br>-And harmonious DIVERSITY of nature<br>-CONTRIVANCE<br>-ADAPTATION (green colour)<br>-WORK (bees')	*Same as 1st ed.*	*Same as 1st ed.*<br>+<br>-ADAPTATIONS<br>-CO-ADAPTATIONS<br>- Really wondrous and beautiful ORGANISATION<br>-MAP (Prof. H. D. Rogers')	*Same as 3rd ed.*<br>+<br>-CRYSTALLINE LENS<br>-ORGANIC BEINGS<br>-OBJECTS (3 times)<br>-VOLUTE and CONE SHELLS<br>-PRODUCTIONS of nature (flowers)<br>-FLOWERS (2 times)<br>-FRUITS<br>-MALE ANIMALS, BIRDS, FISHES, MAMMALS, BUTTERFLIES, INSECTS<br>-MALES	*Same as 3rd ed.*<br>–<br>~~-ADAPTATION (green colour)~~<br>~~-COLOURS~~<br>+<br>-ADAPTATION<br>-ANIMALS	*Same as 5th ed.*<br>–<br>~~-ADAPTATION~~<br>~~-FRUITS~~<br>~~-LIVING OBJECTS~~<br>+<br>-ADAPTATION (green colour)<br>-And curious ADAPTATIONS<br>-And complex STRUCTURE (shoveller-duck)<br>-THE IDEA of what is beautiful<br><br>-MALE ANIMALS, BIRDS, FISHES, REPTILES,

	-Perfect and beautiful ADAPTATION of the larva<br>-Endless FORMS			-COLOURS (2 times)<br>-LIVING OBJECTS		MAMMALS, BUTTERFLIES (see 4th ed.)
OCCURRENCES	15	15	19	34	34	35
BEAUTIFULLY	-PLUMED seed of the dandelion<br>-ADAPTED to its end (structure of a comb)<br>-HOOKED seeds<br>-CONSTRUCTED natatory legs<br>-ADAPTING (power)	*Same as 1st ed.*	*Same as 1st ed.*<br>+<br>-RELATED to complex conditions of life (parts of organic beings)	*Same as 3rd ed.*<br>+<br>-COLOURED (male and female)	*Same as 4th ed.*	*Same as 4th ed.*<br>+<br>-ADAPTED (giraffe's frame)<br>-ADAPTED (structures)
OCCURRENCES	5	5	6	7	7	9
DELICATE	-SHELLS<br>-HEXAGONAL WALLS	*Same as 1st ed.*	*Same as 1st ed.*<br>+<br>-NATURE (quality)<br>-Cell-constructing WORK	*Same as 3rd ed.*<br>+<br>-BRANCHING CORALLINE	*Same as 4th ed.*	*Same as 4th ed.*<br>−<br>~~-Cell-constructing WORK~~<br>+<br>-INHABITANTS of the cells<br>-FILAMENTS<br>-MEMBRANE<br>-TEXTURE<br>-INNER COAT of the eye<br>-Fleshy ORGANS
OCCURRENCES	2	2	4	5	5	10
DELICATELY	-DIFFUSED colour (by the bees)<br>-As delicately as a painter could have done with his brush	*Same as 1st ed.*	*Same as 1st ed.*	*Same as 1st ed.*	*Same as 1st ed.*	*Same as 1st ed.*
OCCURRENCES	2	2	2	2	2	2
**DIVINE**	**-POWER (citing Whewell)<br>-LOVE (bibliography) (2 times)**	***Same as 1st ed.***<br>**+<br>-AUTHOR**	***Same as 2nd ed.***<br>**−**<br>~~**-LOVE (bibliography) (2 times)**~~	***Same as 2nd ed.***<br>**+<br>-ELEMENTS (bibliography)**	***Same as 3rd ed.***	***Same as 3rd ed.***
**OCCURRENCES**	**3**	**4**	**2**	**5**	**2**	**2**
ENTHUSIASTIC	-ADMIRATION (structure of a comb)	*Same as 1st ed.*	*Same as 1st ed.*	*Same as 1st ed.*	*Same as 1st ed.*	*Same as 1st ed.*
OCCURRENCES	1	1	1	1	1	1
EXQUISITE	-ADAPTATIONS<br>-STRUCTURE of a comb	*Same as 1st ed.*	*Same as 1st ed.*	*Same as 1st ed.*	*Same as 1st ed.*	*Same as 1st ed.*
OCCURRENCES	2	2	2	2	2	2

EXQUISITELY	-CONSTRUCTED hooks<br>-ADAPTED parts and organs	*Same as 1st ed.*	*Same as 1st ed.*	*Same as 1st ed.*	*Same as 1st ed.*<br>+<br>-FEATHERED gills	*Same as 5th ed.*
OCCURRENCES	2	2	2	2	3	3
EXTRAORDINARY	-COMBINATION of circumstances<br>-Inherent TENDENCY to vary<br>-FACT (2 times)<br>-CAPACITY<br>-MANNER (Proteolepas' loss of shell)<br>-DEGREE<br>-MANNER (development of a part) (3 times)<br>-AMOUNT of modification (3 times)<br>-SIZE<br>-MANNER (development of an organ)<br>-Well-ascertained FACTS (emphasis)<br>-INSTINCT<br>-CASE of Hippeastrum<br>-ABUNDANCE of the individuals<br>-MANNER (spreading of European productions)<br>-DIFFICULTY	*Same as 1st ed.*<br>+<br>-Shaped PIECES of bone	*Same as 1st ed.*<br>–<br>~~-Shaped PIECES of bone~~<br>+<br>-INSTINCT<br>-FORCE	*Same as 3rd ed.*<br>–<br>~~-FORCE~~<br>+<br>-CASES<br>-MANNER (variation of the eggs of the Australian Bronze cuckoo)	*Same as 4th ed.*<br>–<br>~~-MANNER (variation of the eggs of the Australian Bronze cuckoo)~~<br>+<br>-OUT-GROWTHS<br>-ADAPTATION<br>-DEGREE in colour<br>-DEGREE in the length<br><br>-CASES of Hippeastrum, Passiflora (see 1st ed.)	*Same as 5th ed.*<br>+<br>-MANNER<br>-DEGREE<br>-HABIT of pecking holes<br>-CASES<br>-TYPE<br><br>-MANNER in the length (see 5th ed.)
OCCURRENCES	21	22	23	24	27	32
EXTRAORDINARILY	-ABNORMAL species<br>-RAPID increase of naturalised productions<br>-SEVERE mortality<br>-DEVELOPED part or organ (3 times)<br>-GREAT modification<br>-LONG horns<br>-DIFFERENT<br>-RARE gales of wind<br>-SHAPED pieces of bone	*Same as 1st ed.*<br>–<br>~~-SHAPED pieces of bone~~	*Same as 1st ed.*	*Same as 1st ed.*<br>+<br>-COMPLEX subject	*Same as 4th ed.*<br>–<br>~~-DIFFER in length and form of beak~~	*Same as 5th ed.*

	-DIFFER in length and form of beak					
OCCURRENCES	12	11	12	13	12	12
FORMIDABLE	-	-	-	-	-OBJECTION<br>-BARRIER (sea)	*Same as 5th ed.*<br>+<br>-ARRAY (objections against natural selection)
OCCURRENCES	0	0	0	0	2	3
GORGEOUS	-PLUMAGE (birds of paradise)	*Same as 1st ed.*	*Same as 1st ed.*	*Same as 1st ed.*<br>+<br>-BIRDS	*Same as 4th ed.*	*Same as 4th ed.*
OCCURRENCES	1	1	1	2	2	2
HARMONIOUS	-DIVERSITY of nature	*Same as 1st ed.*	*Same as 1st ed.*	*Same as 1st ed.*<br>+<br>-BEAUTY	*Same as 4th ed.*	*Same as 1st ed.*
OCCURRENCES	1	1	1	2	2	1
HARMONIOUSLY	-	-	-	-BLENDED forms (citing Dr. Seemann)	*Same as 4th ed.*	*Same as 4th ed.*
OCCURRENCES	0	0	0	1	1	1
**HOLY**	**-LAND (3 times) (bibliography)<br>-ALTAR (bibliography)<br>-PLACES (bibliography)**	***Same as 1st ed.***	**-**	***Same as 1st ed.*<br>+<br>-SCRIPTURE (2 times) (bibliography)<br>-LAND (2 times) (bibliography)**	**-**	**-**
**OCCURRENCES**	**5**	**5**	**0**	**9**	**0**	**0**
**IMMATERIAL**	**-For us (2 times)**	***Same as 1st ed.***	***Same as 1st ed.*<br>+<br>-Whether or not...**	***Same as 3rd ed.***	***Same as 3rd ed.*<br>+<br>-Whether or not...**	***Same as 5th ed.***
**OCCURRENCES**	**2**	**2**	**3**	**3**	**4**	**4**
MAGNIFICENT	-Compound EYES (chrysalis stage butterflies)	*Same as 1st ed.*	*Same as 1st ed.*	*Same as 1st ed.*	*Same as 1st ed.*	*Same as 1st ed.*
OCCURRENCES	1	1	1	1	1	1
MAGNIFICENTLY	-	-	-	-COLOURED butterflies	*Same as 4th ed.*	*Same as 4th ed.*
OCCURRENCES	0	0	0	1	1	1
MARVELLOUS	-AMOUNT of diversification<br>-INSTINCT<br>-INSTINCTS<br>-FACT<br>-MANNER (islands of the Galapagos Archipelago	*Same as 1st ed.*	*Same as 1st ed.*	*Same as 1st ed.*	*Same as 1st ed.*<br>–<br>~~-MANNER (islands of the Galapagos Archipelago tenanted by very closely related~~	*Same as 5th ed.*<br>–<br>~~-INSTINCT~~<br>+<br>-Yet not absolutely perfect CHARACTERS

	tenanted by very closely related species)				~~species)~~ + -FACT (tenanted islands of the Galapagos Archipelago)	-CASE of Cecidomyia
OCCURRENCES	5	5	5	5	5	6
MARVELLOUSLY	-	-	-	-PERFECT <u>attributes</u> (the eye)	-PERFECT <u>characters</u> (the eye) (see 4th ed.)	-
OCCURRENCES	0	0	0	1	1	0
**MIRACULOUS**	**-ACT/S of creation (2 times)**	***Same as 1st ed.*** **+ -What is supernatural or miraculous (citing Butler)**	***Same as 2nd ed.*** **+ -INTERPOSITION -PROCESS**	***Same as 3rd ed.***	***Same as 3rd ed.***	***Same as 3rd ed.***
**OCCURRENCES**	**2**	**3**	**5**	**5**	**5**	**5**
**MYSTERIOUS**	**-LAWS of the correlation of growth -CAUSES -The SUCCESSION of the same types of structure... ceases to be mysterious -A MANNER (inhabitants linked together)**	***Same as 1st ed.***	***Same as 1st ed.***	***Same as 1st ed.***	***Same as 1st ed.***	***Same as 1st ed.*** **+ -CASES of correlation**
**OCCURRENCES**	**4**	**4**	**4**	**4**	**4**	**5**
**MYSTICAL**	**-**	**-**	**-NATUR-PHILOSOPHIE (reference to Oken's work)**	***Same as 3rd ed.***	***Same as 3rd ed.***	***Same as 3rd ed.***
**OCCURRENCES**	**0**	**0**	**1**	**1**	**1**	**1**
NICELY	-BALANCED forces (2 times) -BALANCED scale in the struggle for life	*Same as 1st ed.*	*Same as 1st ed.*	*Same as 1st ed.*	*Same as 1st ed.*	*Same as 1st ed.*
OCCURRENCES	3	3	3	3	3	3
PICTURESQUE	-	-	-	-	-BEAUTY in scenery	-
OCCURRENCES	0	0	0	0	1	0
PRETTY	-CLEAR -FREELY	*Same as 1st ed.*	*Same as 1st ed.*	*Same as 1st ed.*	*Same as 1st ed.* –	*Same as 5th ed.*

	-WELL				~~-CLEAR~~	
OCCURRENCES	3	3	3	3	2	2
PRODIGIOUS	-NUMBER of plants<br>-AMOUNT of difference (2 times)<br>-QUANTITY of fluid nectar<br>-DIFFERENCE (between ants)<br>-MOVEMENTS (rocks)<br>-LAPSE of time<br>-VICISSITUDES of climate<br>-GEOGRAPHICAL revolutions	*Same as 1st ed.*	*Same as 1st ed.*	*Same as 1st ed.*	*Same as 1st ed.*<br>–<br>~~-DIFFERENCE (between ants)~~<br>~~-LAPSE of time~~	*Same as 5th ed.*<br>+<br>-LEAPS (monkey's tail)<br>-TRANSFORMATIONS
OCCURRENCES	9	9	9	9	7	9
**SACRED**	**-BEETLE of the Egyptians**	***Same as 1st ed.***	***Same as 1st ed.***	***Same as 1st ed.***<br>**+**<br>**PLACES (bibliography)**	***Same as 1st ed.***	***Same as 1st ed.***
**OCCURRENCES**	**1**	**1**	**1**	**2**	**1**	**1**
SPLENDID	-RESULTS<br>-FRUIT	*Same as 1st ed.*	*Same as 1st ed.*	*Same as 1st ed.*	*Same as 1st ed.*	*Same as 1st ed.*
OCCURRENCES	2	2	2	2	2	2
STUPENDOUS	-DEGRADATION	*Same as 1st ed.*	*Same as 1st ed.*	*Same as 1st ed.*	*Same as 1st ed.*	*Same as 1st ed.*
OCCURRENCES	1	1	1	1	1	1
**SUPERNATURAL**	**-**	**-What is supernatural or miraculous (citing Butler)**	***Same as 2nd ed.***	***Same as 2nd ed.***<br>**+**<br>**-The supernatural (bibliography: part of the content of Guizot's work)**	***Same as 2nd ed.***	***Same as 2nd ed.***
**OCCURRENCES**	**0**	**1**	**1**	**2**	**1**	**1**
SWEET	-PEAS<br>-JUICE (3 times)<br>-EXCRETION	*Same as 1st ed.*	*Same as 1st ed.*	*Same as 1st ed.*	*Same as 1st ed.*<br>–<br>~~-JUICE~~	*Same as 5th ed.*
OCCURRENCES	5	5	5	5	4	4
WONDERFUL	-DIFFERENCE in beaks<br>-DEVELOPMENT<br>-SKILL of gardeners<br>-FACT/S (6 times)<br>-STRUCTURE (the eye)<br>-POWER of scent	*Same as 1st ed.*	*Same as 1st ed.*	*Same as 1st ed.*<br>+<br>-Differing MANNER (offspring of two sexes)<br>-The most wonderful of all CASES (alternate	*Same as 4th ed.*<br>–<br>~~-The most wonderful of all CASES (alternate generations of animals)~~<br>+	*Same as 5th ed.*<br>–<br>~~-STRUCTURE (the eye)~~<br>~~-METAMORPHOSES in function~~<br>+

	-METAMORPHOSES in function<br>-INSTINCT/S (7 times)<br>-Not very wonderful INSTINCTS (by the Melipona)<br>-Not very wonderful modifications of INSTINCTS<br>-Hardly more wonderful INSTINCTS (bird's nesting)<br>-SORT of shield (worker ants)<br>-COLLECTION of fossil bones<br>-RELATIONSHIP (between the dead and the living)<br>-NUMBER of peculiar land-shells<br>-Endless FORMS			generations of animals)	-DIFFERENCE (between worker ants and perfect females)<br>-THICKNESS (sedimentary strata)<br>-CHANGES of structure<br>-LAW of the long endurance of allied forms<br>-FACT	-ORGAN (the eye)<br>-POWERS of the human eye<br>-CHANGES in function<br>-One of the most wonderful ANIMALS in the world (Greenland whale)<br>-MANNER (changing natural species)<br>-CO-ADAPTATIONS<br>-CONNECTING LINK (Typotherium)<br>-CASE/S (2 times)<br>-MANNER in which certain butterflies imitate other species
OCCURRENCES	27	27	27	29	33	41
WONDERFULLY	-LITTLE practice (Mozart at three years old)<br>-PERFECT structure (hive-bee's)<br>-DIFFERED in shape (the working ants' jaws)<br>-SUDDEN (extermination of whole groups of beings)<br>-DEPARTING (degraded flowers, from the proper type of the order)<br>-DIVERSE forms (Crustacea)<br>-COMPLEX jaws and legs in crustaceans	*Same as 1st ed.*	*Same as 1st ed.*<br>–<br>~~-DIVERSE forms (Crustacea)~~	*Same as 1st ed.*<br>+<br>-DISTINCT (forms of Crustacea)<br><br>-COMPLEX jaws and legs of crustaceans (see 1st ed.)	*Same as 4th ed.*	*Same as 4th ed.*<br>+<br>-ALIKE (eyes)<br>-DIFFERING in structure (two kinds of flowers)<br>-LITTLE pollen<br>-CLOSE (resemblance of insects)<br>-PERFECT (prehensile organ)<br>-CLOSE (gradations)<br>-CHANGED (individuals)<br>-FINE (gradations)
OCCURRENCES	7	7	6	8	8	16
WONDROUS	-ORGANS	*Same as 1st ed*	*Same as 1st ed*<br>+<br>-Really wondrous and beautiful ORGANISATION	*Same as 3rd ed.*	*Same as 3rd ed.*	*Same as 3rd ed.*
OCCURRENCES	1	1	2	2	2	2
